# “Beyond the Finish Line” the Epidemiology of Injury and Illness in Professional Cycling: Insights from a Year-Long Prospective Study

**DOI:** 10.3390/sports13010020

**Published:** 2025-01-14

**Authors:** Thomas Fallon, Rory Nolan, John Peters, Neil Heron

**Affiliations:** 1 Centre for Public Health, Queen’s University Belfast, Belfast T12 6BA, UK; n.heron@qub.ac.uk; 2 Edinburgh Sports Medicine Research Network & UK Collaborating Centre on Injury and Illness Prevention in Sport (UKCCIIS), Institute for Sport, PE and Health Sciences, University of Edinburgh, Edinburgh EH8 8AQ, UK; 3 Uno-X Mobility Cycling, NO-0661 Oslo, Norway; 4 Blue Cat Technical Ltd., Greens Court, West Street, Midhurst GU29 9NQ, UK

**Keywords:** injury and illness surveillance, cycling, road cycling, health

## Abstract

**Introduction:** Injury and illness rates within cycling are a growing concern for riders, medical personnel, and event organisers. This study is the first to document injury and illness rates in professional cyclists throughout one competitive season including training and racing. **Methods:** A prospective, longitudinal study was conducted with 47 professional cyclists (30 males and 17 females) over the 2024 season (1 November 2023–31 October 2024). Injuries and illnesses were defined and recorded following the International Olympic Committee (IOC) consensus guidelines for injury reporting in sports and its cycling-specific extension. Data collection utilised a centralised online hub, integrating exposure metrics (e.g., training hours and kilometres) and medical records. All data were processed on a Macintosh computer using the Microsoft Office and R statistics packages epi tools, binom.test function, and ggplot. (V.4.3.2, R Foundation for Statistical Computing, Vienna, Austria). Ethical approval was obtained from Queens University Belfast, number MHLS 23_175. **Results:** Fifty-five injury events were logged, with 1.15 (±0.359) locations injured per incidence and 1.57 (±1.06) injury types per incident. The overall combined injury rate for racing was 4.14 (95% CI: 2.65–5.79) per 1000 h of exposure, with the overall combined rate for training being 1.23 (95% CI: 0.8–1.7) per 1000 h. The injury risk ratio (RR) for injury during racing and training for females was 11.10 (95% CI: 2.69–37.60), and the RR for males was 10.24 (95% CI: 3.84–43.06), both indicating there is a significantly higher risk of injury during racing compared to training. Abrasions were the most common injury type, with fractures being the most burdensome injury. The most common illness was upper respiratory, 0.63 (95% CI: 0.27–0.99) per year for males and 1.11 (95% CI: 0.64–1.59) per year for females. Saddle sores were the second most common at 0.20 (95% CI: 0.04, 0.36) per year for males and 0.08 (95% CI: 0–0.18) per year for females. **Conclusions:** This study provides the first comprehensive, season-long surveillance data for injuries and illnesses in male and female professional road cycling, highlighting the significant differences in injury profiles between racing and training. These results underscore the need for targeted injury prevention strategies and the establishment of a standardised injury and illness framework for professional cycling.

## 1. Introduction

Injury and illness surveillance is viewed as one of the cornerstones of injury and illness prevention across all sports. Indeed, there have been many proposed frameworks for injury prevention, ranging from Van Meechlans’ ‘sequence of prevention’ [1], to Fullers risk reduction framework [2], Finch’s Translating Research into Injury Prevention Practice (TRIPP) [3] framework, and Bolling’s revised contextual “Sequence of Prevention model” [4]. One common theme that runs through all these prevention models is the foundation of injury/illness surveillance. The sport of cycling, particularly road cycling, is challenged in its ability to manage injuries, with professional cyclists, team principals, and race organisers raising concerns about injuries occurring within racing. Prospective epidemiological research within cycling across all competitive cycling is lacking [5,6]. There is only one study within professional road cycling that has prospectively presented injuries throughout a racing season [7], and there are no prospective injury insights of training injuries in road cycling [6].

Injury and illness surveillance is the basis on which epidemiological sports medicine researchers begin to evaluate the injury and illness profiles of sports and establish the prevalence and risk of injury or illness. This enables practitioners to establish what prevention/risk reduction measures can be implemented and the next steps to reduce injury/illness. Many of the top-tier professional sports have organisation-driven surveillance systems in place. In 2001, professional football developed the UEFA Football Safety Project, aimed at improving athletes’ health and reducing injuries, with a specific football injury reporting consensus first developed in 2006 and updated in 2022 [8]. Similarly, rugby models have followed similar epidemiological systems established at both national and international levels [9]. This injury data have led to rule changes to reduce injuries, with a recent example in rugby being the rule changes to tackle height [10]. In football, rule changes [11,12] have been made to heading the ball in the youth game. In 2022, the Scottish Football Association announced that its professional players would be banned from heading the ball the day before and the day after competitive matches. These rule changes have improved the safety of the game and reduced the injury risk and negative impact on long-term health outcomes, with injury surveillance being the means of evaluation [13].

The Union Cycliste Internationale (UCI), the world cycling governing body, has highlighted its mission to “*promote and support research in cycling epidemiology and medicine, especially for the benefit of lesser-known disciplines*” within its 2030 Agenda [14]. The UCI has recently formed SafeR (for SafeRoad cycling), a specialist entity to oversee all aspects of cycling safety [15]. This entity is partly funded by professional road riders themselves, which highlights the point that riders are concerned about the safety of the sport and want action. However, the sport of cycling remains behind other sports in injury epidemiology, with recent editorials supporting the calls for action and the development of injury surveillance programmes within professional cycling [16].

Historically, studies reporting injuries within competitive cycling disciplines lacked methodological guidance and standardised reporting until the publication of the International Olympic Committee (IOC) consensus statement extension for competitive cycling in 2021 [17,18]. The IOC consensus recommends reporting injuries within road cycling per 1000 h (racing hours and training hours), in addition to per 365 athlete days to enable a comparison with other disciplines. In elite and professional road cycling, there is a mix of levels across varying studies which include professional athletes. Studies include professionals only [7,19,20], elite/professional [21,22,23,24,25], and combined amateur, elite, and professional [26]. Furthermore, with regard to the sex in road cycling, there is a mix of studies which included both males and females [7,22,23,24,25,26,27,28,29,30,31] and males only [7,20,21,32,33]. However, to date, no study has presented prospective injury and illness data on male and female professional riders. Since the publication of this IOC consensus, there have been two studies published within the sport that align with its recommendations [7,34]. A recent systematic review examining injuries and illness across all competitive cycling disciplines suggests that the rate of injury within road cycling is 3.68 (1.56–5.45) injuries per year, with all the studies being carried out within racing. Additionally, it suggests that the illness rate is 2.19 (1.62–2.76) per year [5]. However, there has never been a full one-season study which presents injury and illness in cyclists across racing and training. Studies examining injuries in elite cyclists suggest rates of 3.01 per 1000 h of racing [19], with more recent research in professional road racing showing the rate of an injury to be as high as 6.6 per 1000 h [7]. The true incidence of a profile of training-related injuries is poorly understood. Retrospective studies have suggested that knee injuries are the most common overuse injury location, with rates of 0.862 per 365 days [21], with earlier research suggesting the rates were 0.26 per 365 days of training [32]. Therefore, it remains unknown if the injury profile differs between racing and training and how the injury profile between activities compares to other sports.

This study aims to present the injury and illness profile of professional male and female cyclists through one full season. This will be the first study to present prospective injury and illness insights in professional cycling and outline methods that enable such data collection. This study will allow medical professionals responsible for the care of professional cyclists to better understand the injury and illness profile of elite cycling and order the priority of injury/illness prevention steps.

## 2. Methods

### 2.1. Study Design and Participants

The participants of this prospective, longitudinal study were professional road cyclists representing a range of ethnicities training and racing through the 2024 season. The professional cycling season was defined from the 1 November 2023 to the 31 October 2024. A professional cyclist is defined as a cyclist who competes at the national/international level and receives a regular salary or income for their involvement in the sport [5]. The team medical staff provided athletes with detailed information about the proposed study and obtained informed consent from all athletes. This study followed the injury reporting guidelines published in the IOC Consensus on recording and reporting data for injury and illness in sport and the IOC cycling-specific extension [17,18]. Ethical approval for this study was obtained from the Medical Faculty at Queen University Belfast, Northern Ireland (Faculty REC Reference Number: MHLS_23_175).

### 2.2. Injury and Illness Definitions

In cycling, injury and illness are defined in alignment with the IOC consensus on recording and reporting data for injury and illness in sports [17,18]:

1. Injuries are defined as “tissue damage or other derangement of normal physical function due to participation in sports, resulting from rapid or repetitive transfer of kinetic energy requiring medical attention”.
2. Illness is defined as “a complaint or disorder experienced by an athlete, not related to injury. Illnesses include health-related problems in physical (e.g., influenza), mental (e.g., depression) or social well-being, or removal or loss of vital elements (e.g., air, water, warmth)”.

### 2.3. System Design and Data Collection

The hub system is an online purpose-built software new to cycling designed to manage all data aspects of a professional cycling team, such as racing, training, health, equipment, and logistics. This system identifies each athlete within the respective team with a Unique ID (UID). The hub uses a variety of application programming interfaces (APIs) to collate information for each athlete from various sources (official results, training software, medical notes, and meteorological data) into a central server independently managed and hosted on Shiny applications. This central server allows the opportunity to extract accurate integrated datasets relevant to each athlete in the group with little extra administration burden on team medical staff or directors.

The respective team doctor logged each medical contact in the FITSTATS Technologies, Inc. Athlete Monitoring system. This was in a standardised form, as discussed before the commencement of the study. These data were centrally stored with UID on an independently monitored server in compliance with GDPR.

### 2.4. Descriptive and Statistical Analysis

All athlete data were anonymised using a UID and exported into an Excel database (Microsoft Excel 2018, Windows) from the Hub software. Injury and illness data logged throughout the year were downloaded and associated with metrics such as duration (Hrs), distance (KM), and activity type (racing and training), allowing injury and illness incidence rates to be accurately expressed across all exposure denominators.

Injury and illness incidence were calculated separately using the total number of injuries/illnesses in racing and training as the numerator and total exposure as the denominator. All the statistical tests were two-sided, and results with *p* < 0.05 were statistically significant. All data were processed on a Macintosh computer using Microsoft Office and R statistics (V.4.3.2, R Foundation for Statistical Computing, Vienna, Austria). These incidences were multiplied by 1000 and 365 to provide a rate per 1000 h and a rate per 365 days, respectively, to give rates in line with the IOC cycling extension. Equation (1) mean (including 95% CIs) and median severity (days absence) were reported. Injury and illness burden was calculated as the product of injury/illness incidence and mean injury severity and reported as days absence per 1000 h. To assess differences between incidence, severity, and burden in racing and training, incidence risk ratios (RRs) (95% CI) were calculated, as were mean differences (95% CI)—Equation (2) using the epi tools Package in R Statistics. Confidence intervals were calculated using the binom.test function and ggplot function in R.

**Equation (1)**—Injury and Illness Incidence Calculation [Numerator for 365 Days Incidence]:

(1) $$Injuries/Illness\;per\;1000\;h\;\left[365\;Days\right]=\left(\frac{Number\;of\;Injuries/Illness\;}{Total\;Exposure}\;\right)\times 1000[365]$$

**Equation (2)**—Risk Ratio Calculation:

(2) $$RiskRatio=\left(\frac{Injuries/Illness\;incidence\;in\;the\;group\;(A)}{Injuries/Illness\;incidence\;in\;comparator\;Group\;(B)}\;\right)$$

## 3. Results

Forty-seven professional cyclists (17 female and 30 male) were monitored for 366 days, with medical professionals logging each injury event. The mean age for male riders was 26 years (SD = 4, range 20–37 years). For female riders, the mean age was 28 years (SD = 5, Range 20–37 years). The total hours (Hrs:Min:Sec) for male athletes was 6045:34:55 for racing and 21,215:34:32 for training, and, for female athletes, it was 2590:07:21 for racing and 9805:54:00 for training. Male athletes had 1454 racing days, equalling 47.9 (±18.0, range 6–86) race days per year per athlete. Female athletes had 566 racing days, equalling 31.81 (±19.68, range 0–56) race days per year athlete.

There were significant differences in monthly training and racing hours between males and females (training (*p* = 0.0025) and racing (*p* = 0.0007)). Additionally, there were significant differences in the race kilometres and training kilometres (*p* = 0.00090 and *p* = 0.0005). For both male and female athletes, the highest number of training hours occurred in December and January. The highest race hours for males were recorded in July, while, for females, they occurred in May. The greatest number of training kilometres for males was recorded in December and May, while females peaked in December and January (Supplementary Material Table S1).

### 3.1. Injuries Overview

**Figure 1:**
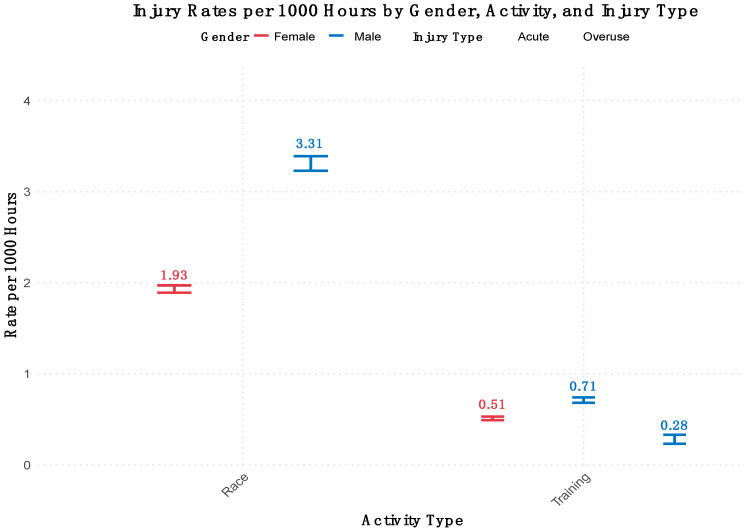
Comparison of injury rate by team, type, and activity for males and females.

Fifty-five injury events were recorded, with 1.15 (±0.359) locations injured per event and 1.57 (±1.06) injury types per event. The overall combined injury rate for racing was 4.14 (95% CI: 2.65–5.79) per 1000 h of exposure and 6.28 (95% CI: 4.02–8.79) per 365 days. The overall combined rate for training was 1.23 (95% CI: 0.8–1.7) per 1000 h. The rate of time-lost injuries for racing is 1.65 (95% CI: 0.66 to 2.81) per 1000 h and 2.51 (95% CI: 1.00–4.26) per 365 days. A breakdown of overall injury rates within training and racing and onset type is presented in Table 1. All overuse (n = 6) injuries occurred in training. The RR for injury during racing and training for females was 11.10 (95% CI: 2.69–37.60), and the RR for males was 10.24 (95% CI: 3.84–43.06), both indicating there is a significantly higher risk of injury during racing compared to training (Figure 1).

There were two cases of Exercise Iliac Artery Endo Fibrosis, 0.05 (95% CI: 0–0.126)/1000 h and 0.042 (95% CI: 0–0.106) per 365 athlete days. Additionally, there was one fractured rib during conditioning and one fractured wrist due to an unknown cause of injury. As the exposure to this environment was unknown, no incident rate was calculated.

The greatest rate of injury in racing for females and males was similar to the greatest rate of injury in racing between January and March. Similar trends were observed with training injuries between sexes, with the greatest rates being seen in April.

#### 3.1.1. Training Injuries

For females, there was an even spread of injuries varying from hand, head, forearm, and wrist. Males had a high incidence of knee injuries, with similar rates of 0.28 (95% CI: 0.10–0.62) per 1000 h (Table 2).

Contusions occurred at 0.10 (95% CI: 0–0.57) per 1000 h in females and 0.05 (95% CI: 0.00–0.26) per 1000 h in males. Fractures were the most common injury type overall for males and females, followed by abrasions and tendinopathy (Table 2).

The most common overuse injury during training was patellar tendinopathy, with a rate of 0.14 injuries (95% CI: 0.03–0.41) per 1000 h. For females, the most significant injury during training is from fractures, 0.20 (95% CI 0.02–0.74) per 1000 h. For males, the injury burden during training injuries was from fractures, 2.19 (95% CI: 0.17–4.21) per 1000 h and lacerations, 0.36 (95% CI: 0–1.37) per 1000 h (Table 2).

#### 3.1.2. Race Injuries

The most common injury types and locations differ between males and females. Abrasions had the highest rate overall for both females and males, with rates of 0.77 (95% CI 0.09–2.79) and 0.99 (95% CI 0.36–2.16) per 1000 h, respectively. For females, head injuries were the most prevalent, with rates of 1.54 (95% CI: 0.42–3.95) per 1000 h. For males, hand and head injuries, particularly fractures and concussions, were the most frequent, with rates of 0.66 (95% CI: 0.18–1.69) per 1000 h. Additionally, the shoulder, elbow, and hip/groin areas are also common injury locations for both genders. Abrasions had the highest rate overall for both females and males, with rates of 0.77 (95% CI 0.09–2.79) and 0.99 (95% CI 0.36–2.16) per 1000 h, respectively (Table 3).

**Figure 2:**
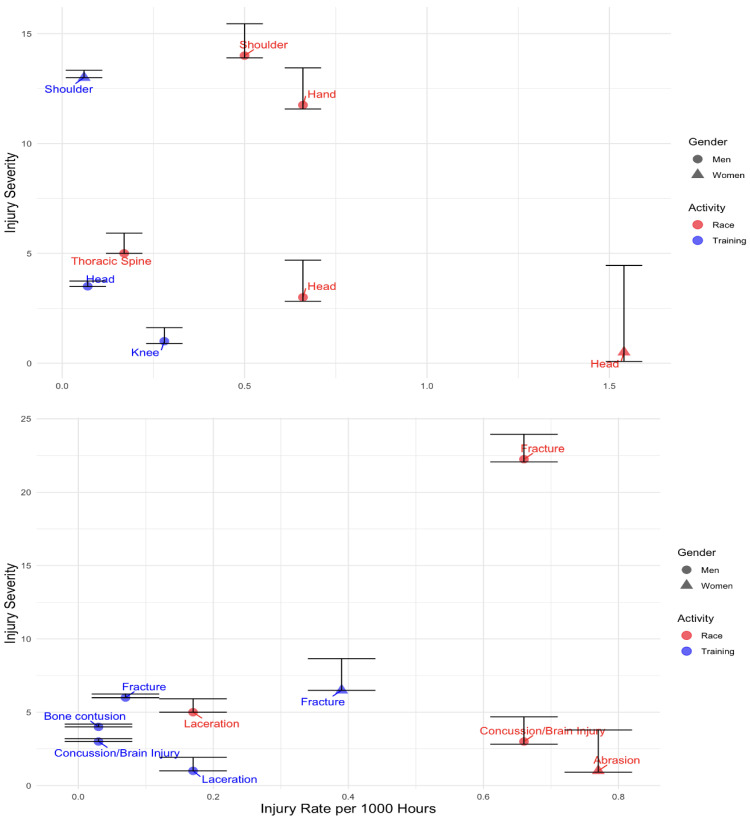
Injury severity (days) per gender for body location and injury type per 1000 h for racing and training.

Male racers experienced the highest burden from fractures at 14.64 (95% CI: 2.14–55.06), followed by hand injuries at 7.75 (95% CI: 2.11–19.83) and shoulder injuries at 7.0 (95% CI: 1.4–20.3) (Figure 2). Among females, training-related fractures had a burden of 2.54 (95% CI: 0–33.74), while race-related abrasions had a lower burden of 0.36 (95% CI: 0–1.37). Head injuries during male races carried a burden of 1.98 (95% CI: 0.57–4.95) (Supplementary Material Table S1).

#### 3.1.3. Illness

**Figure 3:**
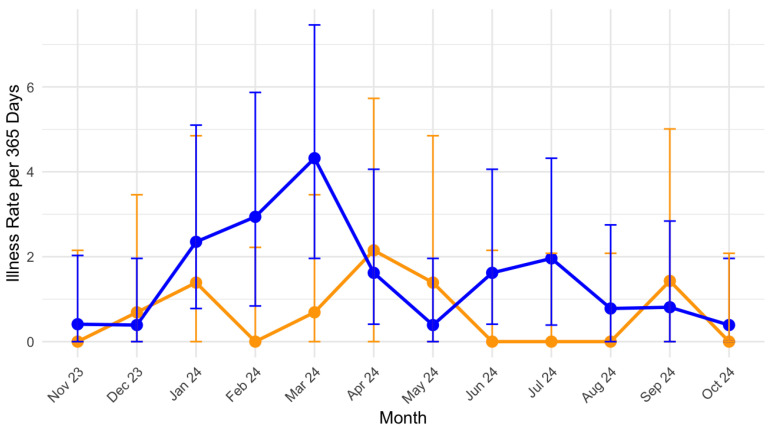
Monthly illness rate per 365 days for males and females. Orange = Female. Blue = Male.

Fifty-six illnesses were logged over the year (Figure 3). Due to the significant difference in exposure times between females and males, our primary measure of illness is per 365 days. The overall illness rate was 1.19 (95% CI: 0.88, 1.5) per 365 days. The rate for females was 0.65 (95% CI: 0.625–0.665) per 365 days, and, for males, 1.46 (95% CI 1.43–1.48) per 365 days. The most affected system was the respiratory system, with a rate in males of 0.80 (95% CI: 0.63–0.96) per 365 days and females of 0.23 (95% CI: 0.16–0.30) per 365 days. The most common type of respiratory illness diagnosis was upper respiratory tract infections (Table 4). For upper respiratory illness, male cyclists had a rate of 0.63 (95% CI: 0.27–0.99) per 365 days, while females had a rate of 1.11 (95% CI: 0.64–1.59) per 365 days. The RR for respiratory illnesses per 365 days between females and males is 1.79 (95% CI: 0.66–4.85). The second most common illness was dermatological, with the main diagnosis being saddle sores: men have a higher rate of 0.20 (95% CI: 0.04, 0.36) per 365 days, compared to females, who have a rate of 0.08 (95% CI: 0–0.18) per 365 days. The greatest burden of illness in females was gastrointestinal, 27.11 (95% CI: 27.01, 27.2) days lost per year. Respiratory illness caused the greatest burden in males with 2.13 (95% CI: 2.11, 2.14) days lost per year.

### 3.2. Discussion Overview

This is the first study to present the injury and illness rates in male and female professional cyclists prospectively throughout a full competitive cycling season. The main points highlighted in this study are as follows:

- Injury rates were significantly higher during racing than training for both genders; the RR for females was 11.10 (95% CI: 2.69–37.60), and, for males, it was 10.24 (95% CI: 3.84–43.06). The combined injury rate during racing was 4.14 (95% CI: 2.65–5.79) injuries per 1000 h versus 1.23 (95% CI: 0.8–1.7) injuries per 1000 h for training.
- Females experienced higher rates of head injuries, 1.54 (95% CI: 0.42–3.95) per 1000 h, in racing, while males had higher rates of fractures and concussions during racing.
- Overuse injuries were exclusive to training with patellar tendinopathy being the most common diagnosis, with 0.14 (95% CI: 0.03–0.41) injuries per 1000 h.
- Fractures and concussions demonstrated the highest severity and injury burden per 1000 h in both training and racing, with males experiencing the greatest burden during races, where fractures had a burden of 14.64 per 1000 h (95% CI: 2.14–55.06) and concussions had a burden of 1.09 per 1000 h (95% CI: 0.05–2.24).
- Upper respiratory tract infections were the most common diagnosis, particularly among females, with rates of 1.11 (95% CI: 0.64–1.59) per 365 days, compared to 0.63 (95% CI: 0.27–0.99) per 365 days for males.
- Saddle sores were more frequently observed in males, with rates of 0.20 (95% CI: 0.04–0.36) per 365 days compared to females, who had a lower rate of 0.08 (95% CI: −0.18–0.18) per 365 days.

#### 3.2.1. Injury

Our findings show the overall rate of injury for racing is 6.28 (95% CI: 4.02–8.79) injuries per 365 days, and training injuries are 0.86 (95% CI: 0.57–1.2) per 365 days. These rates are higher than that proposed in a recent systematic review of injuries and illness in a competitive cycling meta-analysis of 3.65 (95% CI: 1.88–5.47) per 365 days. They are also higher than that observed among professional para-cyclists of 1.94 (95% CI 1.23–2.93) per 365 days.

#### 3.2.2. Training Injury

The training injury incidence of 1.23 (95% CI: 0.8–1.7) per 1000 h is lower than that of soccer, with 7.1 injuries per 1000 h [35] and 3.4 (95% CI: 3.3–3.5) [36], and the 11.8 (95% CI: 11.4–12.2) seen in rugby league [37]. The trend of lower injuries in training in comparison to racing differs from that observed within Down Hill Mountain bikers (DHMTB) and Endro cycling, where higher rates have been seen within training/practice [34,38]. This is likely explained by the discipline-specific differences and the emphasis placed on course familiarisation and skill within DHTMB in comparison to the more endurance-based demands of road cycling, which requires athletes to train for extended periods at relatively low intensity. The lower rates of injury in comparison to field-based sports are understandably explained by the non-contact nature of cycling combined with the fact that cyclists will accumulate a much higher absolute training volume (h) than field athletes.

The low rate of training-related injuries overall, and patella tendinopathy being the most prevalent overuse injury only seen in males, is similar to previous retrospective research [21,32,39]. High training volumes carried out by male athletes may be one factor that explains such injury, which carries no burden to training availability; however, it may impact training volumes during such periods. The unique presentation of External Iliac Artery Endo Fibrosis (EIAE) has been presented in a variety of case studies on cyclists as the cause for the unilateral loss of leg power [40,41,42,43]. EIAE is a flow limitation in the iliac arteries in endurance athletes and is notoriously difficult to diagnose and sometimes career-ending. A commonly used diagnostic measure is a decrease in the ankle-brachial index with flex hips (ABI_Flexed_) following a maximal effort exercise test [44]. Modalities such as an ultrasound can be helpful for imaging in the diagnosis of EIAE, with studies showing that the Doppler measurement of the peak systolic velocity (PSV) is significantly higher in symptomatic limbs provoked by certain movements such as hip flexion [45]. No study in cycling to date has presented information as to the true incidence of this. We present two cases of EIAE which show, whilst relatively uncommon, a low incidence of 0.05 (95% CI: 0–0.126)/1000 h and 0.042 (95% CI: 0–0.106) per 365 athlete days. Medical professionals caring for cyclists should be cognisant of this condition as a cause of unilateral leg pain, which usually requires surgical intervention.

#### 3.2.3. Racing Injury

The rates of injury in racing observed within this study, 3.31 (95% CI 2.02–5.11) per 1000 h, are lower than the 6.5 (95% CI 5.3–8.2) per 1000 h seen within the only prospective racing study at this level [7] for those seen within professional DHMTB [34] and Enduro racers [38]. In comparison to other sports, injury rates are lower than in the rugby union, 91 (95% CI 77–106) per 1000 h [46], and the 23.8 (95% CI 23.2–24.4) per 1000 h seen during matches in men’s football [36]. However, male athletes will race, on average, 328 h per season, which encompasses 6 to 84 race days per season. This is a much higher exposure duration when compared to sports such as football, where it has been shown the mean match hours are 41 h per season [36]. As a consequence of this, the overall rate of injury per 1000 h per season may appear diluted when compared to other field sports per 1000 h. The greatest burden of injury for both males and females was during racing, which suggests that, in addition to the greater risk of injury during racing, these injuries carry a greater burden, particularly for males, with 26.57 (95% CI 16.28–75.43) days lost per year due to race injuries.

When examining the injury profile of cycling, abrasions remain the highest injury type, with 0.99 (95% CI 0.36–2.16) injuries per 1000 h in males and 1.29 (95% CI 0.16–4.66) per 1000 h in females. It should be noted that abrasions often occur as a secondary injury and, despite this rate being high, our study presents only data on the primary injury type for each incident. Steps such as ensuring there is Dyneema^®^ in the cycling kit around the main abrasion sites such as the hip/groin may give some secondary protection against more severe injuries whilst acknowledging the thermoregulatory demands of exercise [47].

Concussions and fractures were the main injury types observed, which is in line with previous systematic reviews in road cycling and prospective studies carried out at this level [6,7]. Our findings show that the head was the second most common injury region, accounting for 14% of all injuries with a concussion rate of 0.66 (95% CI 0.18–1.69) per 1000 h of racing in men and 0.39 (95% CI 0.01–2.15) per 1000 h of racing in females. This is in line with the findings of our recent systematic review of cycling injuries and the rate of head injuries in cycling. Positively, the 9.2% rate of concussion being diagnosed is much higher than the 4.19% (95% CI 3.53–4.96) observed within the systematic review. This is likely due to the increased awareness around sports-related concussions within the peloton after many calls for action [48] and the UCI publication of the Harrogate consensus [49,50] and education cards [51]. However, the diagnosis of sports-related concussion remains an ongoing challenge for those working within cycling [52,53,54]. Our findings show that, despite the rate of concussion being relatively high within racing injuries, the burden of the injury for males is low overall, with 3.00 (95% CI 0.14–6.14) days lost per year due to the injury. Whilst the rate of diagnosis appears to be increasing, concussion remains an ongoing diagnostic challenge. Cycling should trial head accelerometers on helmets, which act as impact detectors for riders in mass crashes, which would help medical professionals identify riders at risk who may have remounted their bike and returned to the peloton before screening. The low number of days lost is not a reflection of the duration for that athlete to return to racing; it is reflective of the duration it took the athlete to commence their grade return to the sport after 48–72 h of rest [55]. Additionally, with the higher rate of head injuries seen within female cycling, which has also been found in other disciplines [34], consideration should be given to neck strengthening to help reduce linear and rotational head accelerations, which commonly cause axonal sheer and concussion symptomology [56,57].

Fractures mostly affected the hand and shoulder and carried the greatest injury burden, with 21.4 (95% CI: 3.12–80.37) days lost per year in males. The protective fall behaviours of cyclists likely explain these injury locations upon crashing. This injury pattern is similar to that observed in previous professional road cycling studies [7,19,32,33]. Low bone mineral density has been seen in cyclists, which may predispose them to increased fracture risk upon crashing [58]. Elite road cyclists often display disordered eating behaviours, which can attenuate low energy availability and, subsequently, low bone mineral density [58]. With this in mind, nutritionists working with cycling should ensure that cyclists have adequate calcium (1000 mg/day) and vitamin D (600–800 IU/Day) levels, in addition to considering collagen supplementation to augment bone collagen synthesis with exercise and increase bone mineral density [58,59]. Physiotherapists and conditioning coaches working with athletes should encourage resistance training or plyometric-based exercises as a mode of mechanical loading of the skeleton to increase bone mineral density [58]. Additionally, cyclists’ perception of weight gain associated with resistance training [60] should be managed, and education should be given, promoting its benefits to both bone health [61] and performance [62].

#### 3.2.4. Illness

Overall, the incidence of illness throughout the year was 1.19 (95% CI: 0.88, 1.5) illnesses per 365 days. Due to the significant differences in training hours between men and women, the rate of illness presented per 365 days is much more reflective, as an illness is not isolated to training or racing. The rate of illness is higher than that seen in women’s 0.7 (95% CI: 0.6 to 0.9) per 365 days [63] and men’s 1.1 (95% CI: 0.6 to 1.8) per 1000 days in football [64]. The findings align with the narrative expectations from the IOC cycling extension paper, suggesting a high rate of respiratory illness among cyclists [17]. Our findings show that female athletes express greater rates of respiratory illness in comparison to males, with the most common illness diagnosis being upper respiratory ones. These findings are in line with those seen at the Pan Am Games [65] and have been highlighted within the IOC respiratory health consensus [66,67]. Prolonged and high-intensity exercise, both of which are pillars of elite cyclists’ training, have been shown to lead to the temporary impairment of immune responses which has more predominant effects on females, which may explain the higher rates seen [68]. It has also been found that grand tour participation is associated with upper respiratory symptoms [69]. The Tour de France happens in July each year, with the Tour de Femmes in the middle of August. Whilst our results show a small spike in illness just after both these periods, the greatest rates of illness are in March for males and April for females. This period coincides with the start of the European racing season for athletes, along with changes in environmental conditions. This change in environmental conditions and intensity of competition has been suggested to potentially accentuate immunosuppression and increase the risk of infection [70]. Ensuring adequate vitamin D supplementation, with an emphasis on hand hygiene and mask-wearing during travel, especially on longer haul travel and load management, is important for reducing such an illness risk [70,71].

Saddle sores, which are classed as a dermatological illness, have been suggested to be a common problem for those within cycling, with recent systematic scoping reviews examining saddle sore prevalence and definitions in male and female athletes [72,73]. Both reviews were unable to present the true prevalence per year. Our findings show that saddle sores were more frequently observed in males, with rates of 0.20 (95% CI: 0.04–0.36) per 365 days, compared to females, who had a lower rate of 0.08 (95% CI: −0.18–0.18) per 365 days. This is potentially explained by males’ significantly higher training and racing volumes in comparison to females, which may increase the risk. Conversely, this may also be linked with female athletes being less likely to disclose saddle sore symptomology, although this has not been researched. Studies have shown that education can be given to riders in the form of published infographics, which may increase the awareness of various mitigation steps that can be taken by riders [74,75].

## 4. Clinical Recommendations

- The use of protective clothing, such as Dyneema^®^-enhanced jerseys, may help reduce the number of abrasion-type injuries and, subsequently, lessen the impact such injury will have on recovery (i.e., broken sleep due to discomfort, infection risk, etc.). These materials are designed to be both lightweight and strong, providing a barrier against skin abrasions. Reducing these injuries should help reduce recovery durations by preventing discomfort, infection risks, and potential scarring, but also improves the athlete’s ability to train and perform without the interruption of long recovery periods.
- Neck-strengthening exercises, particularly for female athletes, may help reduce linear and rotational head accelerations, which predispose athletes towards concussive symptoms and diagnosis. Strengthening the neck muscles helps minimise the linear and rotational accelerations of the head during crashes, lessening the forces that can lead to concussive symptoms. Given the growing awareness around concussions in cycling, these exercises can play a role in mitigating the injury risk in cyclists.
- Bone health should be prioritised through adequate calcium and vitamin D intake and resistance/plyometric exercises to lower fracture risk, with efforts made to address misconceptions about resistance training and potential weight gain. Incorporating resistance and plyometric exercises into training regimens helps improve bone mineral density and overall musculoskeletal health. Addressing misconceptions about resistance training, such as the fear of gaining excessive muscle mass or weight, can encourage cyclists to engage in these beneficial exercises without concerns about their impact on performance.
- Enhanced awareness, early recognition, and adherence to a concussion diagnosis with further discussion are needed on the development of discipline-specific diagnostic considerations. The development of discipline-specific concussion diagnostic frameworks for cycling—taking into account the unique impacts and risks associated with the sport—can improve diagnostic accuracy and treatment, preventing long-term health consequences for riders. Embracing technological advances in the use of head impact accelerometers on helmets feeding back to race doctors’ car may be a positive step toward providing those caring for athletes with an indication for assessment.
- Preventive measures for respiratory illnesses include good hand hygiene, mask-wearing during travel, vitamin D supplementation, and close monitoring during high-risk periods such as early season and grand tours.
- Saddle sore prevention should focus on appropriate saddle fit, education on hygiene practices, and adjustment of training volumes upon early symptoms, particularly in male cyclists. Preventive strategies should include ensuring an optimal saddle fit, which is critical for comfort and avoiding pressure points. Educating cyclists on proper hygiene, such as keeping the area clean and dry and using chamois cream, is essential. Additionally, creating a supportive and open environment for female athletes to disclose symptoms related to saddle sores or discomfort is important, as it encourages early intervention and ensures appropriate care without fear of stigma.

## 5. Future Research Directions

This study provides a foundational understanding of injury and illness rates in one professional cycling team throughout a season. However, a centralised injury surveillance system, similar to those implemented in football and rugby, should be developed for professional cycling to capture injury data, starting with race injury surveillance in the male and female races. This would provide a broader and more detailed understanding of injury patterns within the peloton. This system should include the specific mechanisms of injuries during races and training, incorporating meteorological data, crash dynamics, and terrain types to identify risk factors and high-risk scenarios. This will then allow those working to improve the safety of cycling an objective mechanism of evaluating the effectiveness of proposed preventive measures, such as protective clothing, neck-strengthening exercises, and nutritional interventions, in real-world settings, which would be beneficial in refining these strategies.

## 6. Limitations

As this study was focused on one professional team, the likelihood of all riders crashing in one incident was low. However, within the cycling peloton, there will be between 170–200 riders travelling together with average speeds over 41 Kph. Thus, one rider falling may lead to a domino effect of crashes within the bunch, and, because of this, multiple riders from multiple teams sustain an injury. The methods of this study do not provide such detail. This is an important point to carry forward in further studies, particularly those focused on race injuries, and the UCI should consider a centralised injury surveillance system for race injuries as has been established in sports such as football [36] and rugby [76] for decades [16]. This study did not explore the specific mechanism of injury further than acute, overuse, etc. Further research, particularly that focused on racing, should explore these factors, in combination with meteorological data and time point data from open sources to better understand injury profiles and high-risk points/stages for injury.

## 7. Conclusions

This is the first study to present prospective injury and illness for a group of professional male and female riders. The methods of this study show that prospective IIS can be carried out with little extra administrative burden on medical staff through using the online Hub and Athlete Monitoring software. Injuries, particularly fractures and concussions, are more common during races than in training, with males facing a higher injury burden overall. Females, however, experience a greater frequency of head injuries during racing. Overuse injuries were predominantly seen in training, with patellar tendinopathy being the most common in males.

Illnesses also present a significant challenge, with respiratory infections more prevalent overall. This supports the wider focus being placed on respiratory health issues in elite athletes, particularly during intense competition periods. Gastrointestinal and dermatological illnesses were more common in females, while saddle sores affected more males, possibly due to the higher training and race volumes.

The findings underscore the physical demands of professional cycling, highlighting the need for effective injury prevention strategies, particularly targeting fractures, concussions, abrasions, and respiratory issues. This study also calls for more comprehensive research to better understand gender differences in injury and illness rates and develop tailored interventions to safeguard the health of cyclists and enhance their performance.

## Figures and Tables

**Table 1:** Injury rates per team presented and onset type per 365 days and per 1000 h.

Teams	Activity/ Onset	Count	Rate per 365 Days	Rate per 1000 h	Burden per 1000 h	Burden per 365 Days	Mean Severity (SD)
Male	Training	21	0.7 (0.43–1.07)	0.99 (0.61–1.51)	0.99 (0.61–3.16)	0.7 (0.43–2.24)	1 (±2.09)
Male	Race	20	5.02 (3.07–7.75)	3.31 (2.02–5.11)	17.53 (10.69–49–74)	26.57 (16.28–75.43)	5.3 (±9.73)
Female	Race	5	3.22 (1.05–7.52)	1.93 (0.63–4.5)	0.772 (0.25–4.01)	1.29 (0.42–6.69)	0.4(±0.89)
Female	Training	5	0.29 (0.1–0.68)	0.51 (0.17–1.19)	1.33 (0.44–6.91)	0.75 (0.26–3.95)	2.6 (±5.81)
Male Race	Acute	20	5.02 (3.07–7.75)	3.31 (3.23, 3.39)			
Male Training	Acute	15	0.49 (0.18, 0.80)	0.71 (0.68, 0.74)			
Male Training	Overuse	6	0.16 (0.03, 0.35)	±0.28 (0.23, 0.33)			
Female Race	Acute	5	3.22 (1.05–7.52)	1.93 (1.89, 1.97)			
Female Training	Acute	5	0.29 (0.20, 0.39)	0.51 (0.49, 0.53)			

**Table 2:** Primary training injury type and location with incidence race per 365 training days and 1000 training hours (95% CI in parentheses).

Type Location	Laceration	Contusion (Superficial)	Fracture	Abrasion	Injury Without Tissue Type Specified	Bone Contusion	Tendon Rupture	Concussion/ Brain Injury	Tendinopathy	Muscle Injury	Spinal Cord/Nerve Injury	Female Rate per 365 Days	Female Rate per 1000 h	Male Rate per 365 Days	Male Rate per 1000 h
**Knee**	1			1		1			3			-	-	0.20 (0.07–0.43)	0.28 (0.10–0.62)
**Hand**				1	2		1					0.06 (0–0.33)	0.10 (0–0.57)	0.10 (0.02–0.29)	0.14 (0.03–0.41)
**Head**			1					2				0.06 (0–0.33)	0.10 (0–0.57)	0.07 (0.01–0.24)	0.09 (0.01–0.34)
**Thigh**		1			2							-	-	0.10 (0.02–0.29)	0.14 (0.03–0.41)
**Lower leg**					1					1		-	-	0.07 (0.01–0.24)	0.09 (0.01–0.34)
**Lumbosacral**											2	-	-	0.07 (0.01–0.24)	0.09 (0.01–0.34)
**Abdomen**	1											-	-	0.03 (0.00–0.19)	0.05 (0.00–0.26)
**Elbow**			1									-	-	0.03 (0.00–0.19)	0.05 (0.00–0.26)
**Foot**					1							-	-	0.03 (0.00–0.19)	0.05 (0.00–0.26)
**Forearm**				1								0.06 (0–0.33)	0.10 (0–0.57)	-	-
**Shoulder**			1									0.06 (0–0.33)	0.10 (0–0.57)	-	-
**Wrist**			1									0.06 (0–0.33)	0.10 (0–0.57)	-	-
**Female Rate per 365 Days**	-	-	0.12 (0.01–0.42)	0.06 (0–0.33)	-	-	0.06 (0–0.33)	0.06 (0–0.33)	-	-	-				
**Female Rate per 1000 h**	-	-	0.20 (0.02–0.74)	0.10 (0–0.57)	-	-	0.10 (0–0.57)	0.10 (0–0.57)	-	-	-				
**Male Rate per 365 Days**	0.07 (0.01–0.24)	0.03 (0.00–0.19)	0.07 (0.01–0.24)	0.07 (0.01–0.24)	0.20 (0.07–0.43)	0.03 (0.00–0.19)		0.03 (0.00–0.19)	0.10 (0.02–0.29)	0.03 (0.00–0.19)	0.07 (0.01–0.24)				
**Male Rate per 1000 h**	0.09 (0.01–0.34)	0.05 (0.00–0.26)	0.09 (0.01–0.34)	0.09 (0.01–0.34)	0.28 (0.10–0.62)	0.05 (0.00–0.26)		0.05 (0.00–0.26)	0.14 (0.03–0.41)	0.05 (0.00–0.26)	0.09 (0.01–0.34)				

**Table 3:** Primary race injury type and location with incidence rate per 365 race days and 1000 race hours (95% CI in parentheses).

Type Location	Laceration	Contusion (Superficial)	Fracture	Abrasion	Injury Without Tissue Type Specified	Bone Contusion	Concussion/Brain Injury	Female Rate per 365 Days	Female Rate per 1000 h	Male Rate per 365 Days	Male Rate per 1000 h
**Head**				2	1		5	2.58 (0.7–6.6)	1.54 (0.42–3.95)	1 (0.27–2.57)	0.66 (0.18–1.69)
**Hand**			2			2		-	-	1 (0.27–2.57)	0.66 (0.18–1.69)
**Shoulder**			2	1				-	-	0.75 (0.16–2.2)	0.50 (0.1–1.45)
**Elbow**				2				-	-	0.50 (0.06–1.81)	0.33 (0.04–1.20)
**Hip/groin**				2				-	-	0.50 (0.06–1.81)	0.33 (0.04–1.20)
**Chest**		1						-	-	0.25 (0.01–1.40)	0.17 (0.00–0.92)
**Knee**				1				-	-	0.25 (0.01–1.40)	0.17 (0.00–0.92)
**Lumbosacral**			1					0.64 (0.02–3.59)	0.39 (0.01–2.15)	-	-
**Thigh**		1						-	-	0.25 (0.01–1.40)	0.17 (0.00–0.92)
**Thoracic spine**	1							-	-	0.25 (0.01–1.40)	0.17 (0.00–0.92)
**Wrist**		1						-	-	0.25 (0.01–1.40)	0.17 (0.00–0.92)
**Female Rate per 365 Days**	-	-	0.64 (0.02–3.59)	1.29 (0.16–4.66)	0.64 (0.02–3.59)	-	0.64 (0.02–3.59)				
**Female Rate per 1000 h**	-	-	0.39 (0.01–2.15)	0.77 (0.09–2.79)	0.39 (0.01–2.15)	-	0.39 (0.01–2.15)				
**Male Rate per 365 Days**	0.25 (0.01–1.40)	0.03 (0.00–0.19)	1.00 (0.27–2.57)	1.51 (0.55–3.28)	-	0.50 (0.06–1.81)	1.00 (0.27–2.57)				
**Male Rate per 1000 h**	0.17 (0.00–0.92)	0.05 (0.00–0.26)	0.66 (0.18–1.69)	0.99 (0.36–2.16)	-	0.33 (0.04–1.20)	0.66 (0.18–1.69)				

**Table 4:** Illness system and incidence rates for the top five illness diagnoses (95% CI in parentheses).

System Diagnosis	Ophthalmological	Not Specific	Dermatological	Respiratory	Gastrointestinal	Cardiovascular	Genitourinary	Endocrinological	Multiple Systems or Not Otherwise Specified	Rate per 365 Days Male	Rate per 365 Days Female	Rate per 1000 h Male	Rate per 1000 h Female
Medical Illness/Other		3							1	0.17 (−0.19–0.52)	0.29 (−0.18–0.77)	0.18 (−0.19–0.56)	0.40 (−0.19–0.77)
Other upper resp. tract infection				19					4	0.63 (0.27–0.99)	1.11 (0.64–1.59)	0.70 (0.32–1.07)	1.53 (0.98–1.59)
Saddle Sore			7							0.20 (0.04, 0.36)	0.08 (0–0.18)	0.22 (0.0–0.77)	0.08 (0–0.18)
Viral gastroenteritis					3					0.10 (−0.26–0.46)	0.18 (−0.30–0.65)	0.11 (−0.27–0.46)	0.24 (−0.27–0.65)
Heart arrhythmia/conduction abnormality						2				0.07 (−0.29–0.42)	0.12 (−0.36–0.59)	0.07 (−0.36–0.59)	0.16 (−0.30–0.59)
**Female per 365 Days**	—	0.23 (0.16–0.30)	0.05 (0.02–0.09)	0.23 (0.16–0.30)	0.05 (0.02–0.09)	0.05 (0.02–0.09)	—	—	—				
**Male per 365 Days**	0.06 (0.03–0.09)	0.03 (0.01–0.05)	0.233 (0.14–0.31)	0.800 (0.63–0.96)	0.233 (0.14–0.31)	0.033 (0.01–0.05)	0.03 (0.01–0.05)	0.03 (0.01–0.05)	0.033 (0.01–0.05)				
**Female per 1000 h**	—	0.32 (0.22–0.41)	0.08 (0.03–0.13)	0.32 (0.22–0.41)	0.08 (0.03–0.13)	0.08 (0.03–0.13)	—	—	—				
**Male per 1000 h)**	0.07 (0.041–0.10)	0.03 (0.01–0.05)	0.25 (0.16–0.35)	0.88 (0.70–1.06)	0.25 (0.16–0.35)	0.03 (0.016–0.05)	0.03 (0.01–0.05)	0.03 (0.01–0.05)	0.03 (0.01–0.05)				

## Data Availability

The data that support the findings of this study are available from the corresponding author upon reasonable request.

## Supplementary Materials

The following supporting information can be downloaded at: https://www.mdpi.com/article/10.3390/sports13010020/s1, Table S1: Training and Race injury types and location injury incidence (per 1000 h), severity (days absence) and burden (days absence per 1000 h and 365 days).

## References

[1] van Mechelen W., Hlobil H., Kemper H.C.G. (1992). Incidence, Severity, Aetiology and Prevention of Sports Injuries. Sports Med..

[2] Fuller C., Drawer S. (2004). The Application of Risk Management in Sport. Sports Med..

[3] Finch C. (2006). A new framework for research leading to sports injury prevention. J. Sci. Med. Sport..

[4] Bolling C., van Mechelen W., Pasman H.R., Verhagen E. (2018). Context Matters: Revisiting the First Step of the ‘Sequence of Prevention’ of Sports Injuries. Sports Med..

[5] Fallon T., Heron N. (2024). A systematic review protocol of injuries and illness across all the competitive cycling disciplines, including track cycling, mountain biking, road cycling, time trial, cyclocross, gravel cycling, BMX freestyle, BMX racing, e-sport, para-cycling and artistic cycling. Front. Sports Act. Living.

[6] Rooney D., Sarriegui I., Heron N. (2020). ‘As easy as riding a bike’: A systematic review of injuries and illness in road cycling. BMJ Open Sport. Exerc. Med..

[7] Edler C., Droste J.-N., Anemüller R., Pietsch A., Gebhardt M., Riepenhof H. (2023). Injuries in elite road cyclists during competition in one UCI WorldTour season: A prospective epidemiological study of incidence and injury burden. Physician Sportsmed..

[8] Waldén M., Mountjoy M., McCall A., Serner A., Massey A., Tol J.L., Bahr R., D’Hooghe M., Bittencourt N., Della Villa F. (2023). Football-specific extension of the IOC consensus statement: Methods for recording and reporting of epidemiological data on injury and illness in sport 2020. Br. J. Sports Med..

[9] Moore I.S., Ranson C., Mathema P. (2015). Injury Risk in International Rugby Union. Orthop. J. Sports Med..

[10] van Tonder R., Hendricks S., Starling L., Surmon S., Viviers P., Kraak W., Stokes K.A., Derman W., Brown J.C. (2024). Tackling the tackle 1: A descriptive analysis of 14,679 tackles and risk factors for high tackles in a community-level male amateur rugby union competition during a lowered tackle height law variation trial. J. Sci. Med. Sport..

[11] Duddy C., Adcock A., Woodhouse J. (2023). Debate on a Motion on Football and Dementia.

[12] Iacobucci G. (2021). Dementia risk in professional footballers is linked to player position and career length, study finds. BMJ.

[13] Peek K., Duffield R., Cairns R., Jones M., Meyer T., McCall A., Oxenham V. (2023). Where are We Headed? Evidence to Inform Future Football Heading Guidelines. Sports Med..

[14] Union Cycliste Internationale Agenda 2030. https://assets.ctfassets.net/761l7gh5x5an/6RrOHtU0QlyN80MDJ7vJm3/cf54c913960a66a71baaac379ef12b88/2022_UCI_AGENDA2030_web_EN.pdf.

[15] Union Cycliste Internationale SafeR (for SafeRoad Cycling). https://assets.ctfassets.net/761l7gh5x5an/4rUxp44EcTd8yinslFRNvx/a66c80d850388c3e9fe35b2410f4177b/2021-uci-guide-safety-en.pdf.

[16] Fallon T., Heron N. (2024). Do We Need to Make the Sport of Road Cycling SafeR? Translating Research into Injury Prevention Practice (TRIPP) Frameworkfor Road Cycling. J. Sci. Cycl..

[17] Clarsen B., Pluim B.M., Moreno-Pérez V., Bigard X., Blauwet C., Del Coso J., Courel-Ibáñez J., Grimm K., Jones N., Kolman N. (2021). Methods for epidemiological studies in competitive cycling: An extension of the IOC consensus statement on methods for recording and reporting of epidemiological data on injury and illness in sport 2020. Br. J. Sports Med..

[18] Bahr R., Clarsen B., Derman W., Dvorak J., Emery C.A., Finch C.F., Hägglund M., Junge A., Kemp S., Khan K.M. (2020). International Olympic Committee consensus statement: Methods for recording and reporting of epidemiological data on injury and illness in sport 2020 (including STROBE Extension for Sport Injury and Illness Surveillance (STROBE-SIIS)). Br. J. Sports Med..

[19] Yanturali S., Canacik O., Karsli E., Suner S. (2015). Injury and illness among athletes during a multi-day elite cycling road race. Phys. Sportsmed..

[20] Haeberle H.S., Navarro S.M., Power E.J., Schickendantz M.S., Farrow L.D., Ramkumar P.N. (2018). Prevalence and Epidemiology of Injuries Among Elite Cyclists in the Tour de France. Orthop. J. Sports Med..

[21] Clarsen B., Krosshaug T., Bahr R. (2010). Overuse injuries in professional road cyclists. Am. J. Sports Med..

[22] Engebretsen L., Soligard T., Steffen K., Alonso J.M., Aubry M., Budgett R., Dvorak J., Jegathesan M., Meeuwisse W.H., Mountjoy M. (2013). Sports injuries and illnesses during the London Summer Olympic Games 2012. Br. J. Sports Med..

[23] Soligard T., Steffen K., Palmer D., Alonso J.M., Bahr R., Lopes A., Dvorak J., Grant M.-E., Meeuwisse W., Mountjoy M. (2017). Sports injury and illness incidence in the Rio de Janeiro 2016 Olympic Summer Games: A prospective study of 11274 athletes from 207 countries. Br. J. Sports Med..

[24] Soligard T., Palmer D., Steffen K., Lopes A.D., Grek N., Onishi K., Shimakawa T., Grant M.-E., Mountjoy M., Budgett R. (2022). New sports, COVID-19 and the heat: Sports injuries and illnesses in the Tokyo 2020 Summer Olympics. Br. J. Sports Med..

[25] Edouard P., Dandrieux P.-E., Hollander K., Zyskowski M. (2024). Injuries and illnesses at the Munich 2022 European Championships: A prospective study of 5419 athletes from 52 countries involved in 9 sports. BMJ Open Sport Exerc. Med..

[26] Ueblacker P., Rathmann W., Rueger J.M., Püschel K. (2008). Acute injuries in road bicycle racing. Injury surveillance at the Hamburg UCI ProTour “Cyclassics” 2006. Unfallchirurg.

[27] Decock M., De Wilde L., Bossche L.V., Steyaert A., Van Tongel A. (2016). Incidence and aetiology of acute injuries during competitive road cycling. Br. J. Sports Med..

[28] McLennan J.G., McLennan J.C., Ungersma J. (1988). Accident prevention in competitive cycling. Am. J. Sports Med..

[29] Sillén K., Wallenius V. (2019). Rates and types of injuries during the three consecutive years 2016 to 2018 of the Vätternrundan-One of the world’s largest and longest bicycle races. Traffic Inj. Prev..

[30] Jancaitis G., Snyder Valier A.R., Bay C. (2022). A descriptive and comparative analysis of injuries reported in USA Cycling-sanctioned competitive road cycling events. Inj. Epidemiol..

[31] Roi G.S., Tinti R. (2014). Requests for medical assistance during an amateur road cycling race. Accid. Anal. Prev..

[32] Barrios C., Sala D., Terrados N., Valentí J.R. (1997). Traumatic and Overuse Injuries in Elite Professional Cyclists. Sports Exerc. Inj..

[33] De Bernardo N., Barrios C., Vera P., Laíz C., Hadala M. (2012). Incidence and risk for traumatic and overuse injuries in top-level road cyclists. J. Sports Sci..

[34] Fallon T., Palmer D., Bigard X., Elliott N., Lunan E., Heron N. (2024). ‘Downhill race for a rainbow jersey’: The epidemiology of injuries in downhill mountain biking at the 2023 UCI cycling world championships—a prospective cohort study of 230 elite cyclists. BMJ Open Sport Exerc. Med..

[35] Ekstrand J., Spreco A., Windt J., Khan K.M. (2020). Are Elite Soccer Teams’ Preseason Training Sessions Associated With Fewer In-Season Injuries? A 15-Year Analysis From the Union of European Football Associations (UEFA) Elite Club Injury Study. Am. J. Sports Med..

[36] Ekstrand J., Spreco A., Bengtsson H., Bahr R. (2021). Injury rates decreased in men’s professional football: An 18-year prospective cohort study of almost 12 000 injuries sustained during 1.8 million hours of play. Br. J. Sports Med..

[37] King D.A., Clark T.N., Hume P.A., Hind K. (2022). Match and training injury incidence in rugby league: A systematic review, pooled analysis, and update on published studies. Sports Med. Health Sci..

[38] Palmer D., Florida-James G., Ball C. (2021). Enduro World Series (EWS) Mountain Biking Injuries: A 2-year Prospective Study of 2010 Riders. Int. J. Sports Med..

[39] Borgers A., Claes S., Vanbeek N., Claes T. (2020). Etiology of knee pain in elite cyclists: A 14-month consecutive case series. Acta Orthop. Belg..

[40] Bilman V., Rinaldi E., Sanvito F., Melissano G., Chiesa R. (2021). External iliac artery endofibrosis in an elite female endurance cyclist. J. Vasc. Bras..

[41] Lindner D., Agar G., Domb B.G., Beer Y., Shub I., Mann G. (2014). An unusual case of leg pain in a competitive cyclist: A case report and review of the literature. Sports Health.

[42] Korsten-Reck U., Röcker K., Schmidt-Trucksäss A., Schumacher Y.O., Striegel H., Rimpler H., Dickhuth H.H. (2007). External iliac artery occlusion in a young female cyclist. J. Sports Med. Phys. Fit..

[43] Carfagno V.F., Rouintan J., Rucker M.A., Carfagno D. (2023). External Iliac Artery Endofibrosis: A Discussion on Two Unique Cases. Cureus.

[44] Parks C.A., Chen W.P., Gomez C.G., Labropoulos N., Koullias G., Berman S.S., Leon L.R. (2024). External iliac artery endofibrosis in females: Case reports and review of the literature. Ann. Vasc. Surg.—Brief. Rep. Innov..

[45] Schep G., Bender M., Schmikli S., Wijn P. (2001). Color Doppler used to detect kinking and intravascular lesions in the iliac arteries in endurance athletes with claudication. Eur. J. Ultrasound.

[46] Williams S., Robertson C., Starling L., McKay C., West S., Brown J., Stokes K. (2022). Injuries in Elite Men’s Rugby Union: An Updated (2012–2020) Meta-Analysis of 11,620 Match and Training Injuries. Sports Med..

[47] Naveen J., Jayakrishna K., Sultan M.T.B.H., Amir S.M.M. (2020). Ballistic Performance of Natural Fiber Based Soft and Hard Body Armour- A Mini Review. Front. Mater..

[48] Elliott J., Anderson R., Collins S., Heron N. (2019). Sports-related concussion (SRC) assessment in road cycling: A systematic review and call to action. BMJ Open Sport Exerc. Med..

[49] Heron N., Jones N., Cardwell C., Gomes C. (2023). ‘If in Doubt, Sit Them Out’: How Long to Return to Elite Cycling Competition following a Sports-Related Concussion (SRC)?. Int. J. Environ. Res. Public Health.

[50] Swart J., Bigard X., Fladischer T., Palfreeman R., Riepenhof H., Jones N., Heron N. (2021). Harrogate consensus agreement: Cycling-specific sport-related concussion. Sports Med. Health Sci..

[51] Union Cycliste Internationale (2021). UCI-Concussion Website Information. https://www.uci.org/concussions/61dBdxjPDFHaFu01mnq9N.

[52] McLarnon M., Boyce S., Heron N. (2023). A call to action: The need for concussion assessment and diagnostic protocols for use in the different elite cycling disciplines, including paracyclists. BMJ Open Sport Exerc. Med..

[53] Gomes C., Jones N., Heron N. (2022). Sports-related concussion (SRC) in track cycling: SRC assessment protocol for elite track cycling. BMJ Open Sport Exerc. Med..

[54] Heron N., Elliott J., Jones N., Loosemore M., Kemp S. (2020). Sports-related concussion (SRC) in road cycling: The RoadsIde heaD Injury assEssment (RIDE) for elite road cycling. Br. J. Sports Med..

[55] Patricios J.S., Schneider K.J., Dvorak J., Ahmed O.H., Blauwet C., Cantu R.C., Davis G.A., Echemendia R.J., Makdissi M., McNamee M. (2023). Consensus statement on concussion in sport: The 6th International Conference on Concussion in Sport–Amsterdam, October 2022. Br. J. Sports Med..

[56] Ivanic B., Cronström A., Johansson K., Ageberg E. (2024). Efficacy of exercise interventions on prevention of sport-related concussion and related outcomes: A systematic review and meta-analysis. Br. J. Sports Med..

[57] Elliott J., Heron N., Versteegh T., Gilchrist I.A., Webb M., Archbold P., Hart N.D., Peek K. (2021). Injury Reduction Programs for Reducing the Incidence of Sport-Related Head and Neck Injuries Including Concussion: A Systematic Review. Sports Med..

[58] Hilkens L., van Schijndel N., Weijer V., Boerboom M., van der Burg E., Peters V., Kempers R., Bons J., van Loon L.J.C., van Dijk J.-W. (2023). Low Bone Mineral Density and Associated Risk Factors in Elite Cyclists at Different Stages of a Professional Cycling Career. Med. Sci. Sports Exerc..

[59] Hilkens L., van Schijndel N., Weijer V.C., Decroix L., Bons J., van Loon L.J., van Dijk J.-W. (2024). Jumping Exercise Combined With Collagen Supplementation Preserves Bone Mineral Density in Elite Cyclists. Int. J. Sport Nutr. Exerc. Metab..

[60] Hoon M.W., Haakonssen E.C., Menaspà P., Burke L.M. (2019). Racing weight and resistance training: Perceptions and practices in trained male cyclists. Phys. Sportsmed..

[61] Mathis S.L., Caputo J.L. (2018). Resistance Training Is Associated With Higher Lumbar Spine and Hip Bone Mineral Density in Competitive Male Cyclists. J. Strength Cond. Res..

[62] Vikmoen O., Rønnestad B.R. (2021). A Comparison of the Effect of Strength Training on Cycling Performance between Men and Women. J. Funct. Morphol. Kinesiol..

[63] Amundsen R., Thorarinsdottir S., Clarsen B., Andersen T.E., Møller M., Bahr R. (2024). #ReadyToPlay: Health problems in women’s football–a two-season prospective cohort study in the Norwegian premier league. Br. J. Sports Med..

[64] Serner A., Chamari K., Hassanmirzaei B., Moreira F., Bahr R., Massey A., Grimm K., Clarsen B., Tabben M. (2024). Time-loss injuries and illnesses at the FIFA world cup Qatar 2022. Sci. Med. Footb..

[65] Post E.G., Anderson T., Samson O., Triplett A.N., Gidley A.D., Isono S.S., Watters J., Donaldson A.T., Finnoff J.T., Adams W.M. (2024). High rates of respiratory illnesses upon arrival: Lessons from Team USA at the Santiago 2023 Pan American and Parapan American Games. Br. J. Sports Med..

[66] Schwellnus M., Adami P.E., Bougault V., Budgett R., Clemm H.H., Derman W., Erdener U., Fitch K., Hull J.H., McIntosh C. (2022). International Olympic Committee (IOC) consensus statement on acute respiratory illness in athletes part 1, acute respiratory infections. Br. J. Sports Med..

[67] Schwellnus M., Adami P.E., Bougault V., Budgett R., Clemm H.H., Derman W., Erdener U., Fitch K., Hull J.H., McIntosh C. (2022). International Olympic Committee (IOC) consensus statement on acute respiratory illness in athletes part 2: Non-infective acute respiratory illness. Br. J. Sports Med..

[68] Bauza D.E.R., Silveyra P. (2024). Sex Differences in Exercise-Induced Effects on Respiratory Infection and Immune Function. Respir. Am. Med. J..

[69] Allen H., Price O.J., Greenwell J., Hull J.H. (2021). Respiratory impact of a grand tour: Insight from professional cycling. Eur. J. Appl. Physiol..

[70] Derman W., Badenhorst M., Eken M., Gomez-Ezeiza J., Fitzpatrick J., Gleeson M., Kunorozva L., Mjosund K., Mountjoy M., Sewry N. (2022). Risk factors associated with acute respiratory illnesses in athletes: A systematic review by a subgroup of the IOC consensus on ‘acute respiratory illness in the athlete’. Br. J. Sports Med..

[71] Heron N., Gonzalez J. (2022). Infographic Best practice guideline for treatment of Upper Respiratory Tract Infections (URTIs) in Elite Road Cyclists. J. Sci. Cycl..

[72] Napier D., Heron N. (2022). Getting to the Bottom of Saddle Sores: A Scoping Review of the Definition, Prevalence, Management and Prevention of Saddle Sores in Cycling. Int. J. Environ. Res. Public Health.

[73] Bury K., Leavy J.E., Lan C., O’connor A., Jancey J. (2021). A Saddle sores among female competitive cyclists: A systematic scoping review. J. Sci. Med. Sport.

[74] Napier D.N., Rankin A., Heron N. (2023). Infographic. Getting to the bottom of saddle sores: An infographic. Br. J. Sports Med..

[75] Fallon T., Crowley E., Carragher P. Saddle Sores. A Pain in the Bottom for Cyclists of All Levels. BJSM Blogs. https://blogs.bmj.com/bjsm/2024/07/12/saddle-sores-a-pain-in-the-bottom-for-cyclists-of-all-levels/.

[76] West S.W., Hudson S.J., Starling L., Cross M., Williams S., McKay C.D., Cazzola D., Brooks J.H.M., Murray R., Williams A. (2024). Twenty year analysis of professional men’s rugby union knee injuries from the English premiership shows high rates and burden. Br. J. Sports Med..

