# Supplementary material for: “Beyond the Finish Line” the Epidemiology of Injury and Illness in Professional Cycling: Insights from a Year-Long Prospective Study
Source: Sports (Basel). 2025 Jan 14;13(1):20. doi: 10.3390/sports13010020 (PMC11769022; doi:10.3390/sports13010020)
Supplement: Supplementary file 1 [file sports-13-00020-s001.zip › sports-3385527-Supplementary.pdf]

Supplementary Material Table S1: Training and Race injury types and location injury incidence (per 1000 h), severity (days absence) and burden (days absence per 1000 h and 365 days)

Teams	Activity	Body Area	Burden per 1000 Hours (CI)	Burden per 365 Days (CI)	Mean Severity	SD	Rate 1000hrs	Rate 365 Days
Male	Race	Fracture	14.64 (2.14 - 55.06)	21.4 (3.12 - 80.37)	22.25	10.34	0.66 (0.18 - 1.69)	1.00 (0.27 - 2.57)
Female	Training	Fracture	2.54 (0 - 33.74)	3.71 (0 - 49.24)	6.5	9.19	0.39 (0.01 - 2.15)	0.12 (0.01 - 0.42)
Male	Training	Concussion/Brain Injury	1.09 (0.05 - 2.24)	1.09 (0.05 - 2.24)	3	1.6	0.03 (0.00 - 0.19)	0.05 (0.00 - 0.26)
Male	Race	Concussion/Brain Injury	1.09 (0.51 - 1.68)	3.00 (0.14 - 6.14)	3	0.82	0.66 (0.18 - 1.69)	1.00 (0.27 - 2.57)
Male	Training	Laceration	0.36 (0 - 1.37)	3.00 (1.39 - 4.61)	1	1.41	0.17 (0.00 - 0.92)	0.25 (0.01 - 1.40)
Female	Race	Abrasion	0.36 (0 - 1.37)	1.00 (0 - 3.76)	1	1.41	0.77 (0.09 - 2.79)	1.29 (0.16 - 4.66)
Male	Training	Bone contusion	1.46 (0.17 - 2.75)	1.00 (0 - 3.76)	4	1.8	0.03 (0.00 - 0.19)	0.05 (0.00 - 0.26)
Male	Training	Fracture	2.19 (0.17 - 4.21)	4.00 (0.47 - 7.53)	6	2.83	0.07 (0.01 - 0.24)	0.09 (0.01 - 0.34)
Male	Race	Laceration	1.82 (1.11 - 2.54)	6.00 (0.45 - 11.55)	5	1	0.17 (0.00 - 0.92)	0.25 (0.01 - 1.40)
**Injury Locations**								
Male	Race	Head	1.98 (0.57 - 4.95)	3.00 (0.86 - 7.71)	3	0.82	0.66 (0.18 - 1.69)	1 (0.27 - 2.57)
Female	Race	Head	1.15 (0.12 - 3.58)	1.29 (0.21 - 5.99)	0.5	1	1.54 (0.42 - 3.95)	2.58 (0.7 - 6.6)
Male	Training	Knee	0.28(0.10–0.62)	0.20(0.07–0.43)	1	1.67	0.28 (0.10 - 0.62)	0.20 (0.07 - 0.43)
Male	Race	Thoracic Spine	0.85(0.00–4.60)	1.25(0.05–7.00)	5	2	0.17 (0.00 - 0.92)	0.25 (0.01 - 1.40)
Male	Training	Head	0.24(0.03–0.84)	0.31(0.03–1.19)	3.5	0.71	0.07 (0.01 - 0.24)	0.09 (0.01 - 0.34)
Female	Training	Shoulder	0.78 (0 - 4.29)	1.3 (0 - 7.41)	13	4	0.06 (0 - 0.33)	0.10 (0 - 0.57)
Male	Race	Hand	7.75 (2.11 - 19.83)	11.75 (3.17 - 30.16)	11.75	16.5	0.66 (0.18 - 1.69)	1 (0.27 - 2.57)
Male	Race	Shoulder	7.0 (1.4 - 20.3)	10.5 (2.24 - 30.8)	14	13.11	0.50 (0.1 - 1.45)	0.75 (0.16 - 2.2)
